# Aqueous Extract of Pepino Leaves Ameliorates Palmitic Acid-Induced Hepatocellular Lipotoxicity via Inhibition of Endoplasmic Reticulum Stress and Apoptosis

**DOI:** 10.3390/antiox10060903

**Published:** 2021-06-03

**Authors:** Jen-Ying Hsu, Hui-Hsuan Lin, Charng-Cherng Chyau, Zhi-Hong Wang, Jing-Hsien Chen

**Affiliations:** 1 Department of Nutrition, Chung Shan Medical University, Taichung City 40201, Taiwan; jyhsu0530@gmail.com; 2 Department of Medical Laboratory and Biotechnology, Chung Shan Medical University, Taichung City 40201, Taiwan; linhh@csmu.edu.tw; 3 Research Institute of Biotechnology, Hungkuang University, Taichung City 43302, Taiwan; ccchyau@sunrise.hk.edu.tw; 4 Department of Food Nutrition and Health Biotechnology, Asia University, Taichung City 41354, Taiwan; wangzh@asia.edu.tw; 5 Department of Medical Research, Chung Shan Medical University Hospital, Taichung City 40201, Taiwan

**Keywords:** aqueous extract of pepino leaf, lipotoxicity, endoplamic reticulum stress, apoptosis, oxidative stress, non-alcoholic fatty liver disease

## Abstract

Saturated fatty acid is one of the important nutrients, but contributes to lipotoxicity in the liver, causing hepatic steatosis. Aqueous pepino leaf extract (AEPL) in the previous study revealed alleviated liver lipid accumulation in metabolic syndrome mice. The study aimed to investigate the mechanism of AEPL on saturated long-chain fatty acid-induced lipotoxicity in HepG2 cells. Moreover, the phytochemical composition of AEPL was identified in the present study. HepG2 cells treated with palmitic acid (PA) were used for exploring the effect of AEPL on lipid accumulation, apoptosis, ER stress, and antioxidant response. The chemical composition of AEPL was analyzed by HPLC-ESI-MS/MS. AEPL treatment reduced PA-induced ROS production and lipid accumulation. Further molecular results revealed that AEPL restored cytochrome c in mitochondria and decreased caspase 3 activity to cease apoptosis. In addition, AEPL in PA-stressed HepG2 cells significantly reduced the ER stress and suppressed SREBP-1 activation for decreasing lipogenesis. For defending PA-induced oxidative stress, AEPL promoted Nrf2 expression and its target genes, *SOD1* and *GPX3*, expressions. The present study suggested that AEPL protected from PA-induced lipotoxicity through reducing ER stress, increasing antioxidant ability, and inhibiting apoptosis. The efficacy of AEPL on lipotoxicity was probably concerned with kaempferol and isorhamnetin derived compounds.

## 1. Introduction

Saturated fatty acid is one of the common components in the diet as well as indispensable nutrition for physiological structure and function. Excess lipid in circulation and uptake into cells results in lipotoxicity and contributes to many metabolic disturbances and the progression of non-alcoholic fatty liver disease (NAFLD). Several studies have been reported that lipotoxicity caused insulin resistance, hepatic steatosis, altered gut flora, endothelial dysfunction, and cardiomyopathy [1,2,3]. A clinical study has been reported that the saturated fatty acid contained diet group presented a high blood lipid profile and resulted in the fatty liver increasing palmitate uptake into the liver [4]. Palmitic acid (PA) is a saturated fatty acid as well as one of the free fatty acids circulating in the blood and used as fuel for energy production. An in vitro study reported that PA led to lipotoxicity in hepatocytes and affected lipid homeostasis, endoplasmic reticulum (ER) stress, and apoptosis [5]. In vivo study revealed that mice fed with PA administrated diet exhibited severe liver fibrosis [6].

The lipotoxicity mechanism is concerned with multiple factors including ER stress, oxidative stress, inflammation, and apoptosis. Though ER orchestrates protein, lipid, and sterol homeostasis, lipid accumulation resulted in misfolding or unfolding protein within ER and induced unfolding protein response (UPR) [7]. The change of glucose regulating protein 78 (GRP78) activated by unfolding protein deposition triggered ER stress sensors, IRE (inositol-requiring enzyme 1α) and PERK (protein kinase R-like endoplasmic reticulum kinase), and ATG6 (activating transcription factor 6α) activities and regulated lipid metabolism, redox status, and also cross-linked to apoptosis [3,8]. It was reported that the activity and expression of lipid metabolism-related enzymes, including SREBPs (sterol regulatory element-binding proteins), ACC (acetyl-CoA carboxylase), FAS (fatty acid synthase), and C/EBPs (CCAAT/enhancer binding proteins), were regulated by ER stress sensors and thus aggravated lipid accumulation in hepatocytes [9]. Therefore, a sustained lipotoxic environment deteriorated NAFLD. This led to interest whether a substance that could eliminate the overload saturated fatty acid-induced metabolic stresses could directly inhibit the progression of NAFLD and other metabolic disturbances. 

*Solanum muricatum* Ait is a solanaceous fruit that is natively cultivated in South America, Spain, and Chili. In Taiwan, pepino fruit is cultivated in Peng Hu County, Taoyuan County, and Nantou County. The fruit has been studied for antiinflammation, antiglycation, and cardioprotective effects [10,11]. The phytochemicals in pepino fruit have also been identified as polyphenol-derived compounds [11]. There still are few studies about pepino leaves. Our prior study revealed that aqueous extract of pepino leaves (AEPL) attenuated alcoholic-induced liver injuries and prevented alcoholic fatty liver progression [12]. Moreover, it has been reported that AEPL provides benefits on the metabolic syndrome, including attenuating lipid accumulation, reducing insulin resistance, and declining fasting blood glucose [13]. Both studies mentioned above revealed that AEPL intervention alleviated lipid accumulation in the liver by suppressing lipogenesis as well as by promoting lipid oxidation. In addition, the antioxidant activities, especially enzymatic antioxidants, were increased in AEPL treatment in metabolic syndrome mice for defending oxidative stress.

Herbal medicines contain polyphenol compositions; they were studied with regard to their benefits in reliving lipotoxicity and preventing non-alcoholic steatohepatitis (NASH) aggravation [14]. The polyphenol and flavonoids components of AEPL were studied in the previous study [12], however, to date, the exact chemical compound of AEPL has never been identified. As mentioned earlier, our previous studies have reported on the hepatoprotective effects of AEPL on moderating lipid metabolism and antioxidant response, but the fundamental mechanism of AEPL on lipotoxicity is still unclear. Hence, the present study aimed to investigate the effect of AEPL on hepatic lipotoxicity as well as the identification of the performing chemical compounds, by analyses using liquid chromatography combined with mass spectrometry.

## 2. Materials and Methods

### 2.1. Aqueous Extract of Pepino Leaf (AEPL) Preparation

The extraction from pepino leaves was described previously. Dried pepino leaves were kindly obtained from Peng Hu County, Taiwan. The procedure of extraction was applied as follows: 100 g dried pepino leaves were boiled and extracted in 4 L deionized water. The extraction was filtered and lyophilized to a fine powder. The polyphenol component of each batch of lyophilized powder was measured at a predefined interval to ensure the batch stability. AEPL stock at 10 mg/mL was prepared in PBS and stored at −20 °C in a dark place before use.

### 2.2. Palmitic Acid (PA) Preparation

PA was prepared as described by Yi Luo et al. [15] and Shung Mei et al. [16] studies with slight modification. An amount of 20 mM PA was dissolved in 0.01N NaOH and incubated at 70 °C for 30 min. The fatty acid soap was conjugated with 10% fatty acid-free BSA in PBS to produce 4 mM palmitate-BSA conjugation for stock solution. The PA-BSA conjugation was diluted in a culture medium before use.

### 2.3. Cell Line and Treatment

**Figure 1:**
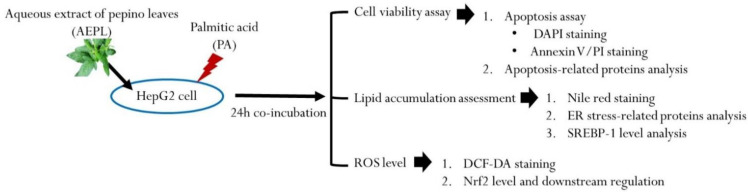
Scheme of experiment design.

The HepG2 human hepatoma cell line was purchased from Bioresource Collection and Research Center (BCRC, Food Industry Research and Development Institute, Hsinchu, Taiwan). The cells were cultured in Minimum Essential Medium supplemented with 10% fetal bovine serum, 0.1 mM non-essential amino acids, 1.0 mM sodium pyruvate, 100 U/mL penicillin, and 100 ug/mL streptomycin in a humidified incubator with 5% CO_2_ at 37 °C. HepG2 cells were seeded at $8\times 10^{5}$ cells/mL in 6-well plates or $2\times 10^{6}$ cells/mL in 10-cm dishes. When the cells’ confluences reached 70–80%, HepG2 cells were treated with 0.3 mM PA in the presence or absence of 5 μg/mL AEPL for 24 h. After incubation, cells were harvested by 0.25% trypsin or scrapper into microtubes for the following experiments. The experiment design in the present study is shown in Figure 1.

### 2.4. Cell Viability Test

$8\times 10^{5}$ cells/mL were seeded to 6-well plates and incubated with different treatments. After 24 h, the cell viability was assessed by staining with 1 mg/mL propidium iodide (Sigma-Aldrich, St. Louis, MO, USA) and analyzed by flow cytometer.

### 2.5. Apoptosis Assay

Cells seeded in 6-well plates were treated with PA (0.3 mM) with/without AEPL (5 μg/mL) for 24 h. Apoptosis in different treatments was measured by the MUSE^TM^ Annexin V and Dead Cell Assay (Lot#MCH100105) according to the manufacturer’s protocol. Briefly, the cell suspension was mixed with an equal volume MUSE^TM^ Annexin V and Dead Cell reagent for 20 min shading from light. The apoptotic rate of each treatment was detected by flow cytometer. 

### 2.6. Reactive Oxygen Species (ROS) Measurement

The ROS level was assessed by using 2′,7′-dichlorofluorescein diacetate (DCF-DA). Cells after treatment were collected in microtubes and stained with 4 μM DCF-DA for 20 min, protecting from light. Fluorescence was detected by flow cytometer. 

### 2.7. Fluorescence Microscopy 

#### 2.7.1. Nile Red Staining

Nile red staining was applied for evaluating lipid accumulation in HepG2 cells with different treatments. After incubating with PA and/or AEPL for 24 h, the medium was removed and cells were fixed with 10% paraformaldehyde for 10 min at room temperature. HepG2 cells were stained with 2 μg/mL Nile red for 15 min. Images were acquired by fluorescence microscopy (Bio-Rad, Hercules, CA, USA) and the area was quantified by ImageJ software.

#### 2.7.2. DAPI (4′-6-diamidino-2-phenylindole) Staining

For assessing the morphological change of apoptosis, DAPI (4′-6-diamidino-2-phenylindole) was applied for nucleus staining. In brief, cells were fixed with 10% paraformaldehyde and stained with DAPI for 30 min at room temperature. After washing with PBS at least three times, nuclear fluorophore in each group was imaged by fluorescence microscopy and quantified by ImageJ software.

#### 2.7.3. Immunofluorescence of HepG2 Cells

HepG2 cells seeded in 6-well plates were fixed with 4% paraformaldehyde for 10 min followed with 0.1% Triton-X-100 for 10 min and then blocked in 5% skim milk for 1 h at room temperature. After blocking, cells were washed at least three times before incubating the primary anti-Nrf2 antibody at 4 °C overnight. Secondary antibody, goat anti-rabbit Alexa Fluor 568 (Life Technologies, Carlsbad, CA, USA), was incubated for 1 h at room temperature. Nuclei were counterstained with DAPI (Sigma-Aldrich, Carlsbad, CA, USA). Images were acquired by fluorescence microscopy.

### 2.8. Mitochondria Isolation

Mitochondria isolation from HepG2 cells was performed by Mitochondrial Isolation kit (Thermo, Rockford, IL, USA) in accordance with manufacturer’s instructions. Cell lysates were homogenized with commercial kit reagents and centrifuged twice for discarding impurities. The mitochondrial pellets were resuspended and the concentration was determined by the BCA protein assay kit.

### 2.9. Protein Extraction and Western Blot Analysis

Cultured cell proteins were lysed by using RIPA lysis buffer and centrifuged for removing impurities. Protein concentrations were quantified by the Dual-Range^TM^ BCA protein assay kit (Energenesis Biomedical Co., LTD, Taiwan). An amount of 20–40 μg protein was loaded and separated in 8–12% SDS-PAGE. Proteins were transferred to nitrocellulose membranes and blocked with 5% skim milk at room temperature for 1 h. After blocking, membranes were washed by TBST at least three times and then incubated in the primary antibody at 4 °C overnight. Antibodies against Bax (sc-526), Bcl-2 (C-2), caspase 3 (sc-373730), cleavage PARP (sc-56196), cytochrome c oxidase IV (COX4, sc-292052), cytochrome c (sc-13156), PERK (sc-377400), and SREBP-1 (sc-13551) were purchased from Santa Cruz Biotechnology (Sacramento, CA, USA). GRP 78 (AF5366), IRE1α (DF7709), phospho- IRE1α (AF7150), and Nrf2 (AF0639) were purchased from Affinity Biosciences (Sacramento, CA, USA). Phospho-PERK (T892) was purchased from ABclonal (Carlsbad, CA, USA). Antibodies were applied according to the manufacturer’s procedures. β-actin was purchased from Sigma-Aldrich and used as an internal control. After incubation, membranes were washed by TBST, followed by incubation of HRP-conjugated secondary antibodies at 4 °C for 1h. Immunoreactive bands were visualized by ECL reagent (Millipore, MA, USA). Blotting bands were detected by the ImageQuantTM LAS 4000 mini (GE Healthcare Bio-Sciences AB, Uppsala, Sweden) device with a digital camera. Protein bands were quantified by ImageQuant TL. The intensity values were normalized to β-actin, which served as an internal control, and then the fold was recalculated with the control group.

### 2.10. Total RNA Extraction and Quantitative Real-Time PCR Analysis

Total RNA was isolated by using TriPure Isolation Reagent. An amount of 1 μg RNA was reversed transcripted to cDNA by SuperScript^TM^ IV Reverse Transcriptase according to the manufacturer’s instructions. Quantitative real-time PCR was performed by PowerUp^TM^ SYBR^TM^ Green Master Mix and detected by StepOne^TM^ Real-Time-PCR system (Applied Biosystems, Foster, CA, USA). Primer sequences are listed in Table 1. Sample Ct value was normalized to ACTB Ct, which was served as an internal control. Quantification of gene expression level was calculated by the 2^−ΔΔCt^ method. 

### 2.11. HPLC/ESI-MS-MS Analysis of Aqueous Pepino Leaf Extract

The HPLC/electrospray ionization (ESI) mass spectrometric analysis of aqueous extract of pepino leaf was performed according to the previous report [10], with minor modification. In brief, the analysis of the prepared extracts was performed using a Waters HSS T3 (2.1 × 150 mm, 1.8 µm, Waters Corp., Milford, MA, USA) analysis column fitted with a Security-Guard Ultra C18 guard column (2.1 mm × 2.0 mm, sub-2 µm, Phenomenex, Inc., Torrance, CA, USA) using an HPLC system consisting of a photodiode-array (PDA) detector. The elution solvent system was performed by gradient elution using two solvents: Solvent A (water containing 0.1% formic acid) and Solvent B (acetonitrile containing 0.1% formic acid). The flow rate during the elution process was 0.2 mL/min and the column temperature was set at 35 °C. The binary gradient elution was conducted as follows: 0–20 min (5–35% B), 20–30 min (35–95% B in 5 min, 30–50 min (95% B isocratic elution) and 50–55 min (95–5% B). The absorption spectra of eluted compounds were scanned within 210 to 600 nm using the in-line diode array detector (DAD) monitored at 254, 280, 325 and 375 nm, respectively. After the compounds were eluted and separated they were further identified with a triple quadrupole mass spectrometer. The system was operated in electrospray ionization (ESI) with both positive and negative ionization modes in a potential of + and −3700 V, respectively applied to the tip of the capillary. 10 μL of prepared sample was directly injected into the column using an autosampler. Nitrogen was used as the drying gas at a flow rate of 10 L/min and the nebulizing gas was set at a pressure of 30 psi. The drying gas temperature was maintained at 325 °C. The fragmentor voltage was 115 V, and the in-source collision induced dissociation (CID) voltage was 15 V. Nitrogen was also used as a collision gas. Quadrupole 1 filtered the calculated *m*/*z* of each compound of interest, while quadrupole 2 scanned for ions produced by nitrogen collision between these ionized compounds in the range of 100–1000 amu at a scan time of 200 ms/cycle. The identification of separated compounds was carried out by comparing their mass spectra provided by ESI-MS and ESI-MS/MS with those of authentic standards when available. The peak area obtained by HPLC-DAD in the scanning range of 210–600 nm for each compound was applied for the quantification of characterized compound with the internal standard method.

### 2.12. Statistical Analysis

All data were expressed as mean ± SD from triplicate independent experiments. Statistical plots and differences were performed by Sigmaplot 12.0 and SPSS 18.0 (SPSS Inc., Chicago, IL, USA) respectively. The comparison within groups was evaluated by one-way analysis of variance (ANOVA) followed by the Student–Newman–Keuls test; *p* < 0.05 was considered as statistically significant. 

## 3. Results

### 3.1. AEPL Reduced PA-Induced Cytotoxicity in HepG2 Cells

**Figure 2:**
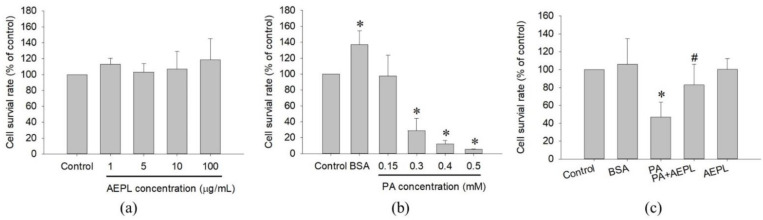
Cell toxicity test of AEPL (**a**) and PA (**b**) in various concentrations. (**c**) The cell viability of 0.3mM PA in presence or absence of 5 μg/mL AEPL for 24 h. All data were presented as mean ± SD of three independent experiments. * *p* < 0.05 vs. control cells; # *p* < 0.05 vs. PA-treated cells.

The study first examined the cytotoxicity of AEPL and PA on HepG2 cells in different concentrations for 24 h treatment. Cell viability results indicated that AEPL in 1, 5, 10, 100 μg/mL had no effect on the survival rate of HepG2 cells (Figure 2a). PA did not affect in 0.15 mM, whereas a higher dose of PA concentration (0.3–0.5 mM) inhibited cell survival more than 50% in a dose-dependent manner at 24 h (Figure 2b). However, 5 μg/mL AEPL treatment to PA-stressed cells significantly increased PA-treated cells viability (Figure 2c). Thus, 5 μg/mL AEPL was adopted for investigating the effect of PA-induced lipotoxicity in HepG2 cells.

### 3.2. AEPL Treatment Altered Lipid Accumulation and Reduced ROS in HepG2 Cell while PA Exposing

**Figure 3:**
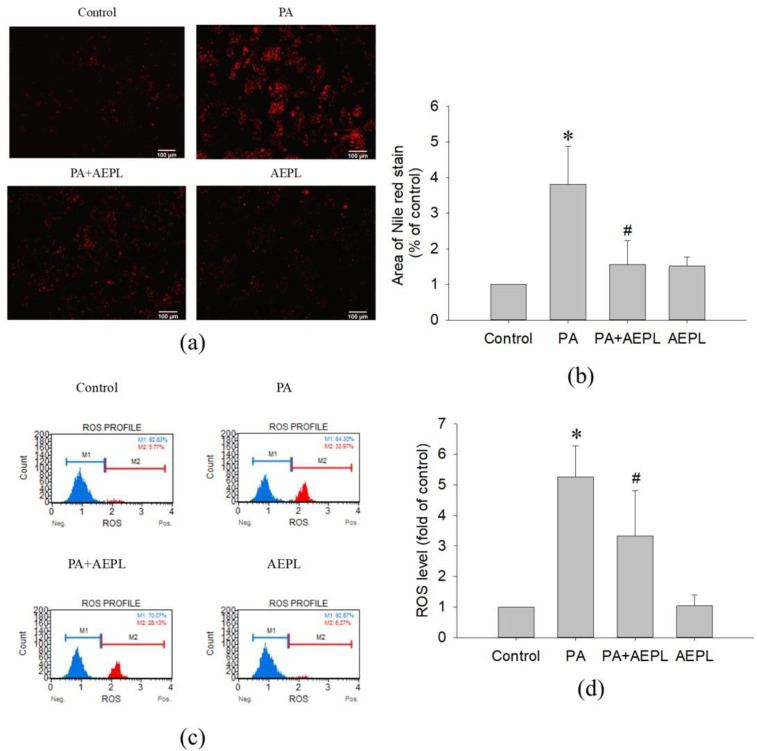
AEPL decreased PA-induced lipid accumulation and ROS production. HepG2 cells were treated with 0.3 mM PA in presence or absence of AEPL for 24 h. (**a**) TG accumulation with different treatment was performed by Nile red staining. Scale bar, 100 μm. (**b**) Quantification of fluorescent area by using ImageJ. (**c**) Intracellular ROS levels were stained with dichlorofluorescin diacetate (DCFH-DA) and analyzed by flow cytometry. M1 indicated DCF-negative cells and M2 indicated DCF-positive cells. (**d**) The percentage of DCF-positive cells were calculated and compared with the control group. All data were performed with three independent experiments. * *p* < 0.05 vs. control cells; # *p* < 0.05 vs. PA-treated cells.

In the Nile red staining, the fluorescence area of PA-treated cells was significantly higher than that of the control cells (Figure 3a,b). The result indicated that PA treatment caused abnormal triglyceride accumulation. While in 5 μg/mL AEPL treatment, the accumulation of triglyceride was significantly reduced (by 80%) in comparison with PA-treated cells (Figure 3b). To evaluate the effect of AEPL on PA-induced oxidative stress generation, HepG2 cells were treated with PA along with/without AEPL for 24 h and analyzed by using flow cytometry (Figure 3c). The ROS level in the PA-treated group significantly increased when compared with the control group (Figure 3d), while AEPL treatment reduced PA-induced ROS level 45% with significance compared with PA group (Figure 3d). These results revealed that triglyceride accumulation and intracellular ROS generation caused by PA were significantly reduced by AEPL.

### 3.3. AEPL Alleviated PA-Induced Apoptosis in HepG2 Cells

**Figure 4:**
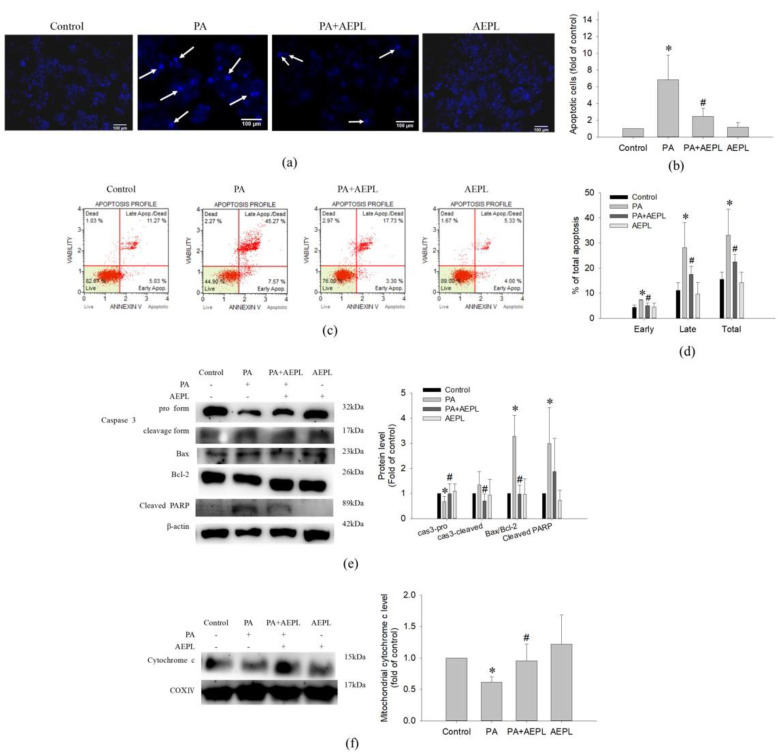
The effect of AEPL on apoptosis. HepG2 cells were treated with PA with/without AEPL for 24 h. (**a**) The morphology change of the nucleus was assessed by DAPI staining. The arrow indicated apoptotic cells. Scale bar, 100 μm. (**b**) Quantification of apoptotic cells by DAPI staining. (**c**) Annexin V/PI binding assay was performed to evaluate the effect of AEPL on PA-stressed apoptotic cells. (**d**) The percentages of apoptotic cells in early, late, and total apoptotic cells were calculated. (**e**) Apoptosis-related proteins (caspase 3, bcl-2, bax, and cleaved PARP) were analyzed by Western blotting. (**f**) Mitochondrial cytochrome c levels were determined by Western blotting and normalized to that of the COXIV. All data were presented as mean ± SD of three independent experiments. * *p* < 0.05 vs. control cells; # *p* < 0.05 vs. PA-treated cells.

To determine the effect of AEPL on apoptosis in PA-stressed cells, we assessed apoptosis by morphology changes and Annexin V/PI staining. After treating PA for 24 h, condensed nuclei could be observed in PA stressed cells in the DAPI staining images, but significantly alleviated in the AEPL treatment group (Figure 4a,b). Annexin V/PI staining was performed for apoptosis analysis. As shown in Figure 4c,d, the proportion of PA-treated cells in both early and late-stage were dramatically increased in comparison with the control cells; this phenomenon was significantly attenuated in AEPL at 5 μg/mL. Next, the apoptosis-related marker proteins were determined by Western blotting. Caspase-3 pro-form and mitochondrial cytochrome c expressions decreased in PA-treated cells in comparison with control cells (Figure 4e,f), whereas these expressions were significantly reduced in AEPL treatment compared with the PA group (Figure 4e,f). The ratio of Bax/Bcl-2 has been implicated as the apoptosis status. The PA-treated group markedly enhanced the ratio of Bax/Bcl-2 but attenuated the ratio by AEPL treatment (Figure 4f). PARP (poly (ADP-ribose) polymerase-1) was studied as it was the substrate of caspase 3 and involved with apoptosis [17]. The change of PARP was assessed in different conditions. Based on the results in Figure 4e, the cleaved form of PARP was increased in the PA-stressed condition. The cleaved PARP level was reduced while co-exposing with AEPL treatment. These results indicated that AEPL treatment suppressed PA-induced apoptosis through inhibiting caspase 3 processing and cytochrome c releasing from mitochondria.

### 3.4. AEPL Reduced ER Stress in PA-Treated HepG2 Cells

**Figure 5:**
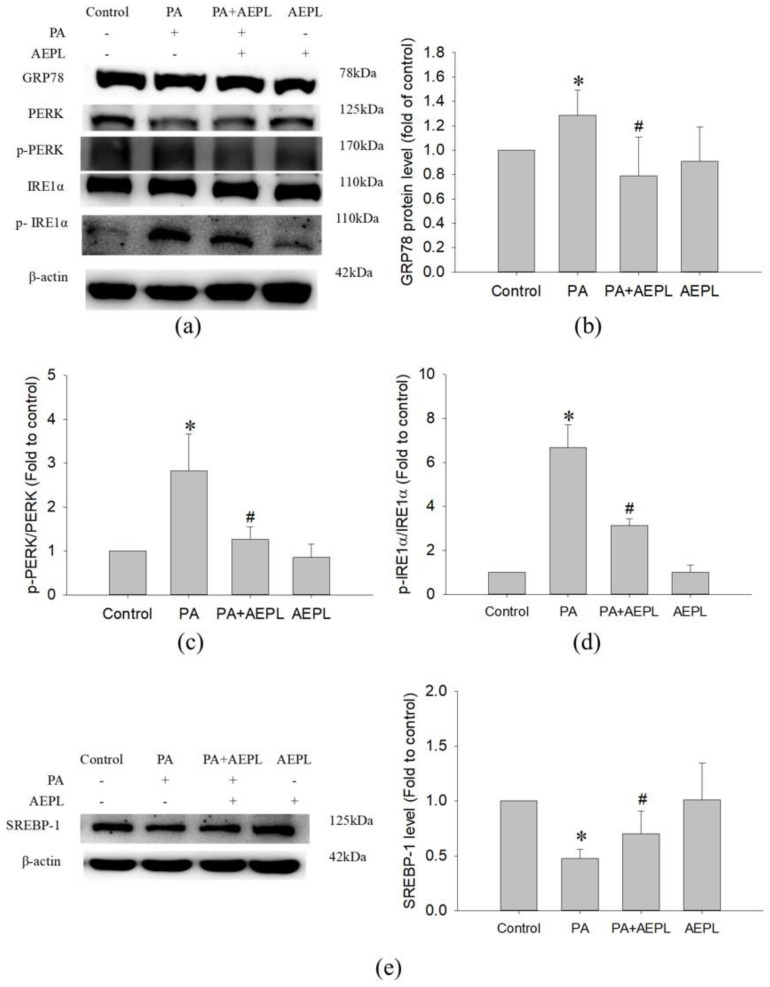
AEPL attenuated ER stress while PA exposure. (**a**) ER stress-related protein levels were analyzed by Western blotting. (**b**–**d**) GRP78, phosphorylated PERK, and phosphorylated IRE1αwere normalized to PERK, IRE1α, and β-actin of each sample. (**e**) SREBP-1 level in each group was normalized to β-actin. All data were presented as mean ± SD of at least three independent experiments. * *p* < 0.05 vs. control cells; # *p* < 0.05 vs. PA-treated cells.

To evaluate the effect of AEPL on the changes of ER stress sensors, including GRP78, IRE1α, and PERK were detected. In the PA condition, GRP78 expression (Figure 5b) and IRE1α activation (Figure 5d) were significantly increased in comparison with control cells. The PERK level in PA stressed cells was lower than in control cells (Figure 5c) which indirectly indicated that PERK phosphorylation and confirmation occurred. Co-existing AEPL markedly decreased the GRP78 level and IRE1α phosphorylation (p-IRE1α) in comparison with the PA group (Figure 5b,d). In addition, the PERK level in AEPL treatment was similar to that of the control cells (Figure 5c). As ER is a critical site of lipid metabolism, the dysfunction of ER altered lipid metabolism. To assess the effect of AEPL on lipogenesis, SREBP-1 expression was detected. As the result in Figure 5e, the precursor form of SREBP-1 was significantly reduced in the PA-treated group compared with the control group. AEPL treatment markedly increased the SREBP-1 level in comparison with PA-treated cells. These results suggested that AEPL treatment alleviated PA-induced ER stress, the response of which was related to lipid accumulation and apoptosis.

### 3.5. AEPL Promoted Nrf 2 Expression and Translocation into the Nucleus

**Figure 6:**
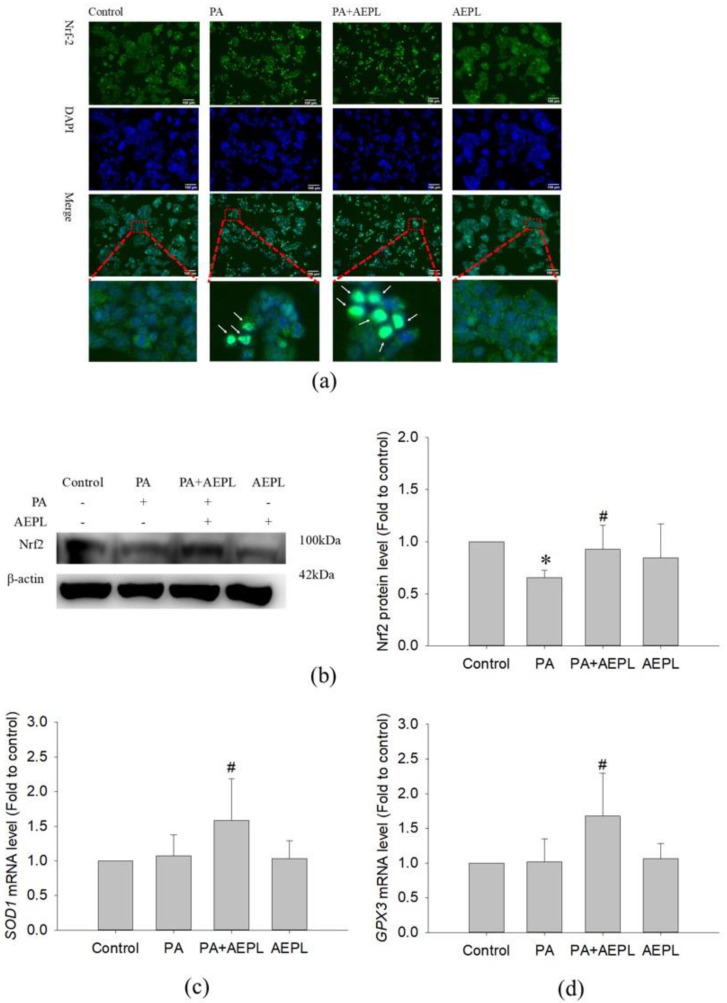
AEPL increased Nrf2 expressions and translocated to nucleus. (**a**) HepG2 cells were treated with AEPL in presence/absence of PA for 24h. Nrf2 localization was determined by using immunocytometry with Nrf2 antibody (green fluorescence). Blue fluorescence was DAPI for staining nucleus. Scale bar, 100 μm. (**b**) Nrf2 protein levels in HepG2 cell lysates by Western blotting and normalizing to that of β-actin. (**c**) The mRNA levels of the *SOD1* gene were normalized to that of the *ACTB* gene. (**d**) The mRNA levels of the *GPX*3 gene were normalized to that of the *ACTB* gene. All data were presented as mean ± SD of three independent experiments. * *p* < 0.05 vs. control cells; # *p* < 0.05 vs. PA-treated cells.

Previous studies have been reported that ER stress, mediated by PERK, elicited Nrf2 activation to respond to intracellular oxidative stress [18,19]. To confirm the effect of AEPL on Nrf2 activity, the translocation of Nrf2 and its protein levels were analyzed. An immunofluorescence assay was performed to detect the translocation of Nrf2. As shown in Figure 6a, Nrf2 was normally localized in the cytoplasm as shown in the control group. However, Nrf2 moved into the nucleus when exposed to PA, whereas AEPL enhanced more cytoplasmic Nrf2 translocation (Figure 6a). Nrf2 was not translocated into the nucleus in normal condition (Figure 6a). The protein level of Nrf2 significantly decreased in PA-treated cells when compared with control cells. However, Nrf2 expression was markedly increased 79% in AEPL treatment in comparison with PA treated cells (Figure 6b). 

To confirm whether the target gene of Nrf2 had been expressed, the mRNA level of *SOD1* and *GPX3* were detected. The changes of *SOD1* and *GPX3* mRNA levels had no effect in PA-treated cells (Figure 6c,d), while co-existing of AEPL, *SOD1* and *GPX3* mRNA level were significantly increased in comparison with control cells and PA treated cells (Figure 6c,d). Collecting the results, in the PA condition, AEPL enhanced the Nrf2 protein level and translocated into the nucleus to promote its target genes *SOD1* and *GPX3* transcription to elevate antioxidant ability.

### 3.6. Identification and Quantification of Phytochemical Constituents of AEPL by HPLC-ESI-MS/MS

**Figure 7:**
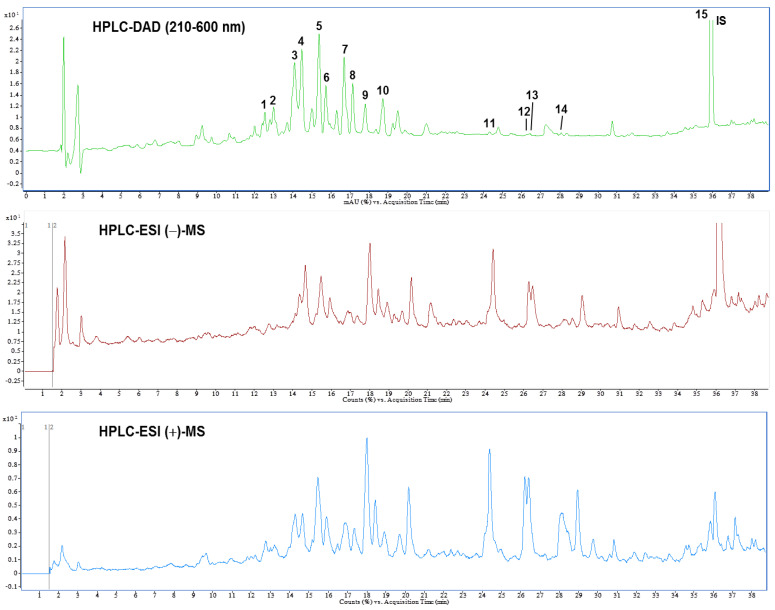
High performance liquid chromatogram detected at a full UV-Vis spectrum of 210–600 nm (top), and total ion chromatograms of negative (middle) and positive (bottom) ionization mass spectrometry from pepino leaf extracts.

A previous study reported the composition of AEPL total phenolic acid, flavonoids, and anthocyanins [12]. The present study identified the chemical composition of AEPL by HPLC-DAD analysis (Figure 7 (top)). According to the UV-Vis spectra, most peaks were centralized in 210–660 nm, which indicated that the compounds included flavonoids moiety. Further, the glycoside conjugation form of flavonoids was identified; the LC-MS and MS/MS were utilized in negative and positive ionization modes. The chromatography and identified compounds are revealed in Figure 7 (middle and bottom) and Table 1. According to Table 2, kaempferol and isorhamnetin-derived compounds are abundant in AEPL. In addition, traces of oxylipins-derived compounds are also identified and quantified in AEPL (Table 2).

## 4. Discussion

Since the liver plays a pivotal role in lipid homeostasis, overload lipid deposition in the liver caused NASH, which further progressed to NAFLD [7]. The present study designed an in vitro model using PA-stressed HepG2 cells for investigating the effect of AEPL on lipotoxicity. PA-induced HepG2 cells were observed in lipid accumulation, increasing ROS level, ER stress, and apoptosis, but prevented by AEPL treatment. According to the results, we suggested that AEPL attenuated PA-induced hepatic steatosis by reducing ER stress and regenerating mitochondria function. In addition, decreasing cleavage of caspase 3 in AEPL treatment indicated the rescue from PA-induced hepatic cell death which drove to NASH aggravation. The intervention of AEPL could attenuate lipid accumulation in the liver, moreover, it could protect lipotoxicity by reducing ER stress and apoptosis.

In this study, AEPL treatment to PA-stressed cells significantly increased the viability of the PA-treated cells (Figure 2c). The mechanisms of hepatocytes viability are complex and are thought to be influenced by the balance between various cell death modes, mainly apoptosis, and by cellular regeneration [28]. The morphology and apoptosis-related proteins analysis implicated that PA-induced apoptosis involving a mitochondrial-mediated pathway has occurred in the PA stressed condition and coincided well with the increase in cell viability as evidenced by the results of Figure 2c, indicating that AEPL might downregulate cell apoptosis to attenuate the damaging effects of PA. Mitochondrial-mediated apoptosis, also known as the intrinsic pathway, is concerned with the stability of mitochondria regulated by Bcl-2 proteins family. A previous study revealed that the cell death resulting from PA-induced lipotoxicity was concerned with the mitochondrial dysfunction [29]. The existence of the apoptosis signal disturbed the interplay of Bcl-2 and Bak/Bax and thus formed the apoptosis pore in mitochondria, resulting in mitochondrial membrane permeabilization [30]. The cytochrome c releasing from mitochondria formed apoptosome with Apaf-1 and caspase 9 whereby initiating the execution phase with series caspase cascade [31]. However, AEPL treatment decreased the PA-induced bax/bcl-2 ratio, which implied that mitochondrial integrity was preserved, thereby retaining cytochrome c in mitochondria which, in turn, avoided caspase 3 activation (Figure 4e,f). Though PARP was the substrate of activated-caspase 3, the cleavage of PARP failed to repair DNA and accelerated apoptosis [17,32]. The change of cleavage PARP form increased exposing of PA (Figure 4e). AEPL treatment in PA stressed HepG2 cells and declined the cleavage form of PARP, which suggested that the activity of caspase 3 was inhibited and ceased the further progression of apoptosis. As regards cellular regeneration, it would be interesting to validate the regenerative mechanism of cell cycle regulation of the extract upon PA administration and is, thereby, needed to be explored in the future. Therefore, we cannot rule out the possibility of AEPL resorted hepatocytes lipotoxicity by inducing cellular regeneration, in addition to inhibiting apoptosis and quenching ROS through its antioxidant properties; future detailed experiments will test this possibility.

ER is a critical organelle for protein processing, especially in the liver, which plays a pivotal role in lipid homeostasis. PA-induced ER stress in hepatocytes was widely acknowledged. Yuren Wei et al. evidenced that hepatocytes with exposing PA impeded ER function and thus resulted in ER stress and apoptosis [33]. Consistent with the previous study, the present study observed that GRP 78 level increased and observed the phosphorylation of IRE1α and PERK in HepG2 cells under the PA-stressed condition (Figure 5b). AEPL treatment restored the ER homeostasis. Our results found that AEPL restored GRP 78 to the normal level and reduced IRE1α and PERK phosphorylation. IRE1α and PERK underwent conformational change and phosphorylation while releasing from GRP78, the phosphorylated state of which regulated downstream signaling pathways, including apoptosis, lipogenesis, and aggravated lipotoxicity [7,34]. SREBP-1 is a lipid sensor transcription factor that resides in ER. When there is low lipid content in cells, SREBP-1 is cleaved and released from ER into the nucleus to promote lipogenesis gene expression. Previous studies have been evidenced that PERK and IRE1α-XBP1 pathway mediated hepatic lipogenesis through upregulating SREBP-1 [35,36]. In addition, the cleavage caspase 3 also regulated SREBP-1 activation, independent of the intracellular lipid level [37] which caused further insults upon the liver. In this study, AEPL restored SREBP-1 precursor form in PA exposing (Figure 5e), which indicated that ER hemostasis was recovered and the lipogenesis was prevented to avoid exacerbating lipid deposition in cells. In addition, to reduce lipogenesis, this study revealed that AEPL treatment preserved the integrity of mitochondria which is associated with its function. As mitochondria are a major organelle of lipid degradation, previous studies reported that mitochondrial dysfunction associated with lipid accumulation [38,39]. Consistent with our prior study observed in metabolic syndrome mice, the current in vitro study demonstrated that AEPL co-exposing with PA in HepG2 cells regulated ER stress to preserve SREBP-1 in precursor status, moreover, restored mitochondria function to decrease lipid accumulation.

AEPL exerted antioxidant ability in alcoholic-induced liver injury mice and metabolic syndrome mice. Oxidative stress-induced by fatty acid, especially PA, which occurred in the liver, contributed to insulin resistance, inflammation, impaired mitochondria, and ER function—all of which formed a vicious cycle [40]. In addition to lipid β-oxidation producing ROS by mitochondrial electron transport, PA-induced ER stress and mitochondrial dysfunction were aggravated by oxidative stress and led to initiate the apoptosis process [41,42]. As consistent with previous studies, ROS overproduction was observed in PA stressed HepG2 cells whereas it declined in AEPL treatment. In the PA stressed condition, AEPL increased the Nrf2 protein level, moreover, the translocation of Nrf2 from cytoplasm to the nucleus was observed. Since Nrf2 is an important sensor of oxidative stress, imbalanced intracellular oxidative stress drove Nrf2 translocation into the nucleus and bound to its promoter region for promoting antioxidant-related gene transcription [43]. It was reported that phosphorylated PERK modulated Nrf2 [19]. The current study suggested that AEPL scavenged ROS through facilitating Nrf2 moving into the nucleus and upregulated its target genes, *SOD1* and *GPX3*, expressions. In addition, the present study identified the flavonols and flavone composition in AEPL. The antioxidant ability of flavonoids derived from plants has been widely studied on reducing oxidative stress by directing quenching ROS [44], inhibiting ROS production [45], and restoring or enhancing antioxidant enzyme activities [46,47]. The results and information suggested that AEPL reduced oxidative stress not only through reducing ER stress and regenerating mitochondrial function but also by driving the Nrf2-dependent antioxidant response. In addition, the flavonoids contained in AEPL acted as ROS scavengers to reduce ROS caused by PA.

The present study is the first to analyze the chemical composition of AEPL. To the best of our knowledge, the current work is the first report on the identification of flavonoids compositions in AEPL. As shown in Table 2, AEPL is mainly composed of kaempferol and isorhamnetin derived compounds, which are considered to contribute to their biological properties on hepatic lipotoxicity protection. The pharmacological effects of kaempferol and its glycosides have been widely studied. According to a study by Yu Wang et al., kaempferol-3-O-glucoside administration protected tetrachloromethane-induced liver damages [48]. *Erica multiflora*, which was abundant in kaempferol-3-O-glucoside, revealed anti-inflammation as well as antioxidant activity in metabolic syndrome mice [47]. Kaempferol, one of the pharmacokinetic metabolites of kaempferol-3-O-glucoside, suggested protected pancreatic β cell function from PA-induced lipotoxicity by regulating AMPK/mTOR pathway to alleviate ER stress [49]. The biological activity of isorhamnetin-3-O-glucoside was explored attenuating oxidative stress, protecting from carbon tetrachloride-induced liver damage, and antidiabetics [38,42,43]. Inconsistent with these studies, the protective effects of AEPL in PA-induced lipotoxicity were probably concerned with these flavonoids derivatives in AEPL. In other words, it may be possible the compounds in the AEPL have different effects to regulate many different intracellular pathways against hepatic lipotoxicity. Future study will be needed to demonstrate that a single, pure component of AEPL has a hepatic lipotoxicity protective effect and to reveal the possible mechanism of action of these compounds, respectively. In addition, the flavonoid metabolites by enterohepatic recirculation and the correlation with pharmacological effects have been discussed in many studies. Hence, the underlying regulation or pharmacological effects of these compositions as well as the metabolites in AEPL is required for further investigation in our future work.

The findings in the current study could be related to previous observations in metabolic syndrome mice. The previous study revealed that AEPL intervention improved lipid profile and recovered lipid homeostasis in the liver in metabolic syndrome mice [13]. To study whether AEPL ameliorates insulin resistance, hyperlipidemia, and hyperglycemia, a high-fat diet combined with low dose streptozotocin (HFD/STZ)-induced metabolic syndrome mouse models treated with AEPL (1%), metformin, or vehicle was set up. Following a 12-week HFD and intraperitoneal injection of STZ, AEPL treatment caused changes in AMPK activation and SREBPs precursor, both of which affected the activities of lipogenesis regulatory enzyme, such as ACC and FAS. The AEPL treatment also promoted β-oxidation which indicated that the stability of mitochondria where β-oxidation takes place. Furthermore, AEPL used in the mouse model to protect from saturated fatty acid-induced lipotoxicity through reducing ER stress, increasing antioxidant ability, and inhibiting apoptosis have not yet been analyzed in full detail. Rebecca C. Rabinovitch et al. have revealed that the activation of AMPK is correlated with mitochondrial function [50]. Collecting the information, we speculated that AEPL probably regulates mitochondria as well as ER to protect from overloading lipid-induced liver damages in vivo. The anti-lipotoxic effect of AEPL on this mouse model validates the cytoprotective mechanism and is, thereby, needed to be explored in the future.

The present study revealed that AEPL clarified the protective mechanism in hepatocytes from PA-induced lipotoxicity, however, the progression of NAFLD evokes an innate immune response and activates Kupffer cells, monocyte-derived macrophages, as well as stellate cells to accelerate inflammation [51]. The interactions of hepatocyte, macrophages and other immune cells are difficult to mimick by using an in vitro model. Moreover, enterohepatic recirculation is a critical metabolism of the bioavailability of flavonoids. The investigation of enterohepatic recirculation is assessed by in vivo study and using bile duct cannulation study [52,53], which cannot be performed by using in vitro experiment system. The pharmacokinetic characteristics of AEPL and the immune response within immune cells in the liver require to be considered for exploring the protective effects on lipotoxicity, hence, there is a limitation in the in-vitro model.

AEPL treatment revealed the protective effects on lipotoxicity in hepatocytes in the present study. In our prior study with the metabolic syndrome animal model, AEPL intervention improved hyperlipidemia and prevented abnormal lipid accumulation as well as increased antioxidant enzyme activity in the liver. AEPL also improved abnormal lipid accumulation in the liver by suppressing lipogenesis, at the same time, promoting β-oxidation [13]. For defending oxidative stress induced by the metabolic stress, AEPL treatment of metabolic syndrome mice elevated enzymatic antioxidant activities, moreover, reducing NAPDH oxidase (unpublished data). The data in the present study clarified the regulatory mechanisms of AEPL on ER, mitochondria, and Nrf2 when co-exposing with PA. These findings demonstrated that AEPL could be a novel dietary supplement or complementary medicine for humans at high risk of metabolic syndrome or NAFLD, for preventing disease progression.

## 5. Conclusions

In summary, the present study demonstrated that AEPL treatment in PA-induced lipotoxicity reduced ER stress, oxidative stress, and prevented cell apoptosis. ER stress was reduced by AEPL regulated SREBP-1 activation as well as increased redox status against oxidative stress-induced cell injury. AEPL reduced PA-induced apoptosis by maintaining the balance of proapoptotic and antiapoptotic proteins as well as mitochondria function. The present study investigated novel insights of AEPL which was protected from PA-induced lipotoxicity and supposed to be a potent nutraceutical of ER stress-reducing agent in the management of NAFLD. However, the pharmacological effect of ingredients in AEPL is required further study.

## Figures and Tables

**Table 1:** Primer sequence of the target genes for real-time PCR.

Gene	Accession No.	Sequence
*SOD1*	NM_000454	Forward: 5′- TAGCGAGTTATGGCGACGAA -3′
		Reverse: 5′- AGTCTCCAACATGCCTCTCTT -3′
*GPX3*	NM_002084	Forward: 5′- AGAAGTCGAAGATGGACTGCC -3′
		Reverse: 5′- CTGGTCGGACATACTTGAGGG -3′
*ACTB*	NM_001101	Forward: 5′- CTGGAACGGTGAAGGTGACA -3′
		Reverse: 5′- AAGGGACTTCCTGTAACAATGCA -3′

**Table 2:** Retention time (t_R_), UV-Vis and ESI Mass spectral characteristics of isolated components in pepino leaf extracts.

Peak	t_R_ (min)	λ_max_ (nm)	[M+H]^+^	[M-H]^−^	MS^2^ (% Base Peak)	Compound	Content (mg/g Extract) ^c^
1	12.77	222sh, 256, 352	-	**595 ^a^**	300(100), 301(8), 271(6)	Quercetin-3-*O*-hexose-*O*-pentoside [20]	2.75 ± 0.04
2	13.22	216, 268, 326	433	**431**	311(100), 283(19), 341(7)	Vitexin [21]	3.15 ± 0.05
3	14.29	266, 330	581	**579**	284(100), 285(74), 255(6)	Kaempferol 3-*O*-xylosylglucoside [22]	8.59 ± 0.15
4	14.69	214, 270, 358sh	611	**609**	315(100), 314(79)	Isorhamnetin-*O*-pentosyl hexoside [23]	19.44 ± 0.45
5	15.19	242, 346	**449**	447	287(100)	Kaempferol-3-*O*-glucoside [24]	28.70 ± 0.16
6	15.93	224, 252, 350	479	**477**	314(100), 285(20), 271(15), 243(13)	Isorhamnetin-3-*O*-glucoside [25]	9.98 ± 0.06
7	16.88	222, 266, 342	535	**533**	284(100), 285(99), 255(19)	Kaempferol-3-*O*-malonylhexoside [20]	20.71 ± 0.70
8	17.37	222, 254, 352	**565**	519	317(100), 107(7)	Isorhamnetin-7-O-(6”-O-malonyl)-glucoside [26]	12.44 ± 0.73
9	18.02	238, 308	327	**651 ^b^**	239(100), 281(39), 227(7)	Unknown	8.68 ± 0.70
10	18.92	220, 292, 316	258	**283**	241(100), 195(29), 223(11), 163(7)	Unknown	12.60 ± 0.59
11	24.38	222	351	**327**	211(100), 229(45), 171(35), 183(12)	oxo-dihydroxy-octadecenoic acid [27]	0.56 ± 0.01
12	26.18	224	353	**329**	211(100), 171(66), 229(65), 139(29)	trihydroxy-octadecenoic acid [27]	0.27 ± 0.01
13	26.38	224	351	**327**	171(100), 201(17), 137(14), 119(13)	oxo-dihydroxy-octadecenoic acid isomer [27]	0.56 ± 0.05
14	28.92	224	353	**329**	201(100), 171(89), 127(27), 139(18)	trihydroxy-octadecenoic acid isomer [27]	0.08 ± 0.03
15	36.01	232, 274, 312	**255**	345	151(100)	7-Methoxyflavanone (IS)	-

^a^ Values in bold indicate the molecular ion for MS/MS fragmentation. ^b^ Pseudo-molecular ion: [2M-H]^−^. ^c^ The values are mean ± standard deviation of HPLC analyses performed in duplicate.

## Data Availability

Data sharing is not applicable.

## Abbreviations

NAFLD	non-alcoholic fatty liver disease
PA	palmitic acid
ER	endoplasmic reticulum
UPR	unfolding protein response
GRP78	glucose regulating protein 78
IRE1α	inositol-requiring enzyme 1α
PERK	protein kinase R-like endoplasmic reticulum kinase
ATG6α	activating transcription factor 6α
SREBPs	sterol regulatory element-binding proteins
ACC	acetyl-CoA carboxylase
FAS	fatty acid synthase
C/EBPs	CCAAT/enhancer binding proteins
NASH	non-alcoholic steatohepatitis
PARP	poly (ADP-ribose) polymerase-1
Nrf2	nuclear factor erythroid 2–related factor 2
